# Delayed Graft Function Under the Microscope: Surveillance Biopsies in Kidney Transplantation

**DOI:** 10.3389/ti.2022.10344

**Published:** 2022-03-24

**Authors:** João Batista Saldanha De Castro Filho, Jeferson De Castro Pompeo, Rafael Berlezi Machado, Luiz Felipe Santos Gonçalves, Andrea Carla Bauer, Roberto Ceratti Manfro

**Affiliations:** ^1^ Division of Nephrology, Hospital de Clínicas de Porto Alegre, Porto Alegre, Brazil; ^2^ UFRGS Medical School, Federal University of Rio Grande do Sul (UFRGS), Porto Alegre, Brazil; ^3^ Division of Transplantation, Hospital de Clínicas de Porto Alegre, Porto Alegre, Brazil

**Keywords:** renal transplantation, delayed graft function, renal biopsy, acute rejection, immunosuppression

## Abstract

Delayed graft function (DGF) is a common complication of kidney transplantation and frequently leads to the necessity of surveillance biopsies. The purpose of this study is to describe the histological findings in surveillance biopsies of deceased donor kidney transplant recipients and evaluate the risk factors for graft outcomes. This is a monocentric, retrospective study including kidney transplant recipients that underwent a graft biopsy during the DGF period between January 2006 and July 2019. 356 biopsies were performed in 335 deceased donor transplant recipients. Biopsies were analyzed according to the Banff classification. The main histological findings were: acute tubular necrosis in 150 biopsies (42.1%), acute rejection in 96 biopsies (26.9%), and borderline findings in 91 biopsies (25.5%). In the multivariate analysis, recipient age (*p* = 0.028) and DGF duration (*p* = 0.005) were associated with rejection, antibody-induction with anti-thymocyte globulin (ATG) was protective (*p* = 0.001). The occurrence of rejection was associated with lower death-censored graft survival (log-rank; *p* = 0.009). Surveillance biopsies of kidney grafts experiencing DGF remain an essential tool for the care of kidney transplant recipients. The recipient’s age and duration of DGF are independent risk factors for acute rejection, while antibody-induction therapy with ATG is associated with protection from its occurrence.

## Graphical Abstract

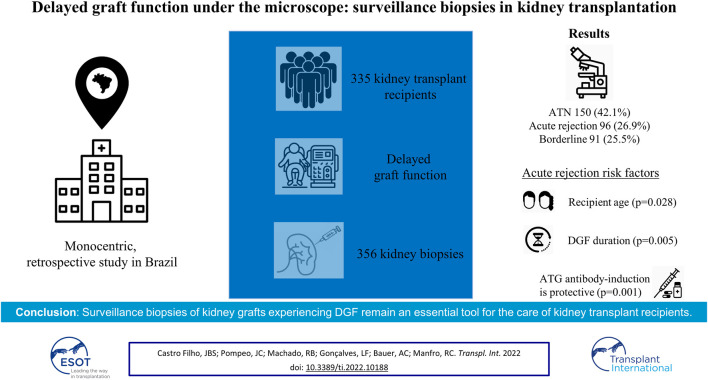

## Introduction

Over the last decades, kidney transplantation became an effective lifesaving procedure for a substantial portion of patients with end-stage kidney diseases (1). Besides increasing life expectancy, successful renal transplants offer a better quality of life than renal replacement therapies (2). Between 2010 and 2019, the number of kidney transplants increased by 35% in Brazil. That occurred mostly due to the increment in deceased donor transplantation since the number of living donor transplants is progressively decreasing in this country (3). As compared to transplants from living donors, deceased donor kidney transplantation is associated with a higher incidence of delayed graft function (DGF), which by itself is associated with acute rejection, lower graft survival, and possibly lower patient survival (4, 5).

Delayed graft function is currently most frequently characterized by the need for dialysis within the first week after transplantation (6). It occurs in approximately one-fourth of kidney transplants in Europe and North America but in Brazil, its incidence is much higher (7-9). The increasing age of the deceased donors, the use of organs from expanded criteria donors (ECD), or with high kidney donor profile index (KDPI), which are usually allocated to older recipients, may contribute to increasing its incidence (10, 11). Other known risk factors include prolonged cold ischemia time, type of preservation solution, preservation technique (static versus pulsatile), and the immunosuppressive regimen (12).

During DGF graft injuries may go unnoticed due to the absence of graft functional parameters used for their monitoring and currently, the only reliable diagnostic tool in this setting is the graft surveillance biopsy. Moreover, the incidence of acute rejection is substantially higher in kidney grafts undergoing DGF (13, 14).

Current transplant guidelines recommend graft tissue histologic evaluation every 7–10 days until the graft acquires function (15, 16). However, such recommendations were made in an era in which the effectiveness of immunosuppressive regimens for the prevention of acute rejection was substantially lower than nowadays (15-17).

The present study aimed to evaluate the utility of surveillance biopsies in uncovering graft injuries, other them those related to ischemia and reperfusion, that would lead to specific treatments, mainly acute cellular rejection and antibody-mediated rejection. We also evaluated the influence of the initial immunosuppressive regimen on the incidence of acute rejection in the surveillance biopsy and patient and graft survivals.

## Materials and Methods

### Study Design, Biopsies and Definitions

**FIGURE 1:**
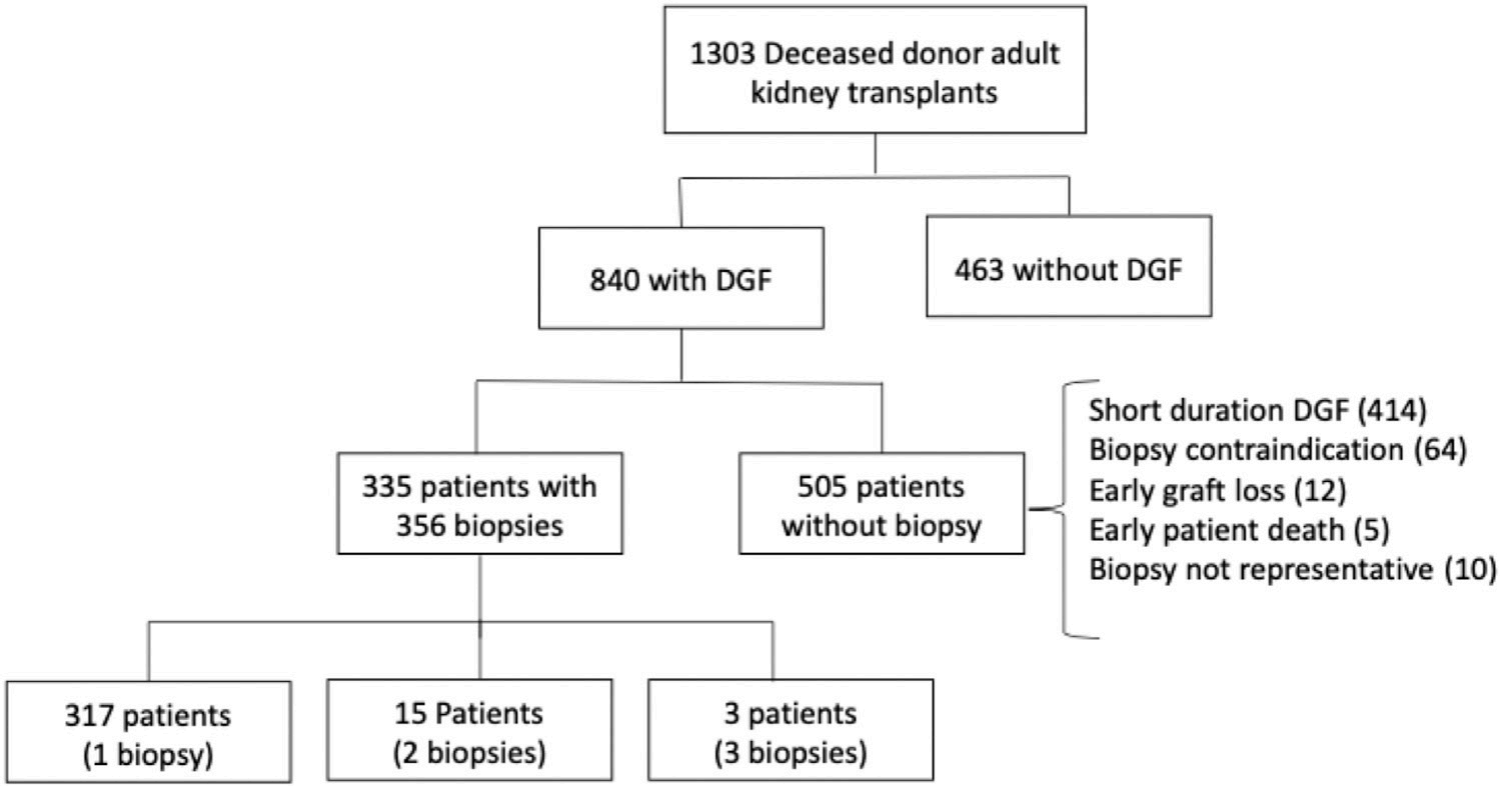
Study outflow.

The study included all adult kidney transplant recipients who received a deceased donor graft, developed DGF, and underwent a surveillance biopsy between January 2006 and July 2019 at Hospital de Clínicas de Porto Alegre, RS, Brazil. We excluded kidney-pancreas and kidney-liver transplant recipients and kidney transplants performed after another solid-organ transplantation. The study flowchart is shown in Figure 1. Data were collected through the review of transplant charts and electronic medical records. Donor, recipient, and transplant-related variables were included for analysis.

During the study period, 1,303 brain dead deceased donor kidney transplants were performed and the vast majority of these organs (1,300) were preserved by cold storage. Three hundred and thirty-five patients underwent 356 representative surveillance biopsies and were included in the study. Kidney allograft biopsies were performed at the attending team’s discretion every 7–14 days during DGF. Biopsies occurred under real-time ultrasonography guidance, through a semiautomatic gun with a 16-G biopsy needle. A renal pathologist analyzed slides stained with hematoxylin-eosin, periodic acid shift, and Masson’s trichrome and interpreted them according to the Banff classification in effect at the time of assessment.

All patients received corticosteroids, calcineurin inhibitors, and mycophenolate as immunosuppressive therapy. Patients that did not receive antibody induction and patients treated with Basiliximab received immunosuppressive drugs at the usual initial doses. Patients treated with anti-thymocyte globulin (ATG) induction, at standard immunological risk, did not receive calcineurin inhibitors until the graft achieved function. Those at high immunological risk received an initially reduced dose. Cellular rejections were treated with corticosteroid pulses or ATG if scored Banff 2A or higher, antibody-mediated rejections were treated with plasmapheresis and polyclonal IV immunoglobulins. Treatment of patients with borderline findings on the surveillance biopsy was decided by the attending team based on the estimated risk of rejection.

Delayed graft function was defined by the requirement of at least one dialysis session during the first week after transplantation (6, 13). DGF duration was recorded from the day of transplantation to the day of the last dialysis session. Expanded criteria donors were defined according to the UNOS criteria (18, 19).

The study was approved by the Ethics and Research Committee of the Hospital de Clínicas de Porto Alegre approved the study (protocol number 64239617.4.0000.5327). The clinical and research activities being reported are in accordance with the ethical standards of the Declaration Helsinki and Declaration of Istanbul on Organ Trafficking and Transplant Tourism.

### Statistical Analysis

Data is presented in absolute numbers, percentages, and frequencies. Continuous variables are presented as mean ± standard deviation, compared using ANOVA, with Tukey post hoc test. Categorical variables are presented as frequencies and compared using Chi-square tests. Sixteen variables were included in the risk analysis for acute rejection. Namely, donor and recipient age, donor and recipient ethnicity, recipient gender, donor final serum creatinine >1.5 mg/dl, expanded criteria donor (ECD), previous transplantation, panel reactive antibodies (PRA) > zero, HLA mismatches, DSA, cold ischemia time, vascular anastomosis time, DGF duration (days), positive historic B cell and presence and type of antibody induction (no induction, Basiliximab, or ATG). In the univariable analysis, prevalence ratio (PR) and 95% confidence intervals (CI) were calculated and the Chi-square test was used to assess their significance. Variables with a *p*-value ≤ 0.2 in the univariable analysis were included in the multivariable analysis model. For the multivariable analysis, prevalence ratios and confidence intervals were estimated by Poison’s regression with robust estimation of variance. We used Kaplan-Meier estimate tests for analyzing patients and grafts survivals and GraphPad Prism for data presentation (version 8; GraphPad Software, San Diego, CA, United States). A *p*-value lower than 0.05 was considered statistically significant. Statistical analysis was performed using SPSS version 18.0 software (SPSS, Inc., Chicago, IL, United States).

## Results

### Study Population Characteristics

In the study period, 356 representative allograft biopsies were performed in 335 transplant recipients. Data of the recipients, donors, and transplant-related variables are shown in Table 1.

**TABLE 1:** Demographic data of recipients, donors and transplants.

Patients/biopsies (number)	335/356
Donor age (years, mean ± SD)	43.7 ± 16.6
Donor creatinine (mg/dl, median; IQR)	1.40 [0.90–2.20]
Expanded criteria donors (number; %)	116 (34.4%)
Recipient age (years, mean ± SD)	46.1 ± 12.9
Male recipient (number, %)	205 (61.2%)
Recipient ethnicity (Caucasian, number, %)	251 (74.9%)
HLA mismatches (ABDR, median; IQR)	3.00 [3.00–4.00]
Panel reactive antibodies (PRA, I and/or II)	
PRA 0 (both class I and II)	164 (48.9%)
PRA 1–50 (either class I or II)	115 (34.3%)
PRA > 50 (either class I or II)	56 (16.7%)
Donor specific antibodies (yes/no)	57 (18.4%)/253 (81.6%)
Cold ischemia time (hours, mean ± SD)	25.6 ± 5.6
Vascular anastomosis time (minutes, mean ± SD)	27.7 ± 6.6
DGF duration (days, mean ± SD)	26.4 ± 19.9
Dialysis sessions (median; IQR)	8.0 [5.00–13.00]
Transplant number ([1, >1]; number, %)	308 (91.9%/27 (8.1%)
Biopsy postoperative day (mean ± SD)	14.7 ± 8.2
First biopsy postoperative day (*N* = 317; mean ± SD)	12.4 ± 6.1
Second biopsy postoperative day (*N* = 15; mean ± SD)	22.6 ± 7.7
Third biopsy postoperative day (*N* = 3; mean ± SD)	31.6 ± 14.3

DGF, delayed graft function; SD, standard deviation; 95% CI, 95% confidence interval; PRA, panel reactive antibodies; IQR, interquartile range.

The majority of the patients were male (61.2%), Caucasian (74.9%), and almost half were not HLA sensitized (48.9% zero PRA). All patients received an initial immunosuppressive regimen with steroids, calcineurin inhibitors (tacrolimus or cyclosporine), and anti-proliferative agents (azathioprine, mTOR inhibitors, or sodium/mofetil mycophenolate) with or without antibody induction. One hundred and forty-eight patients (44.1%) received monoclonal anti-α-chain IL-2 receptor antibodies (Simulect^®^) and underwent 157 biopsies, 151 (45.1%) patients received ATG (Thymoglobulin^®^) and underwent 157 biopsies. Thirty-six patients did not receive antibody induction and underwent 42 biopsies. The most frequent maintenance regimen was tacrolimus, sodium mycophenolate, and steroids in 288 (85.9%) patients.

### Biopsy Results

**FIGURE 2:**
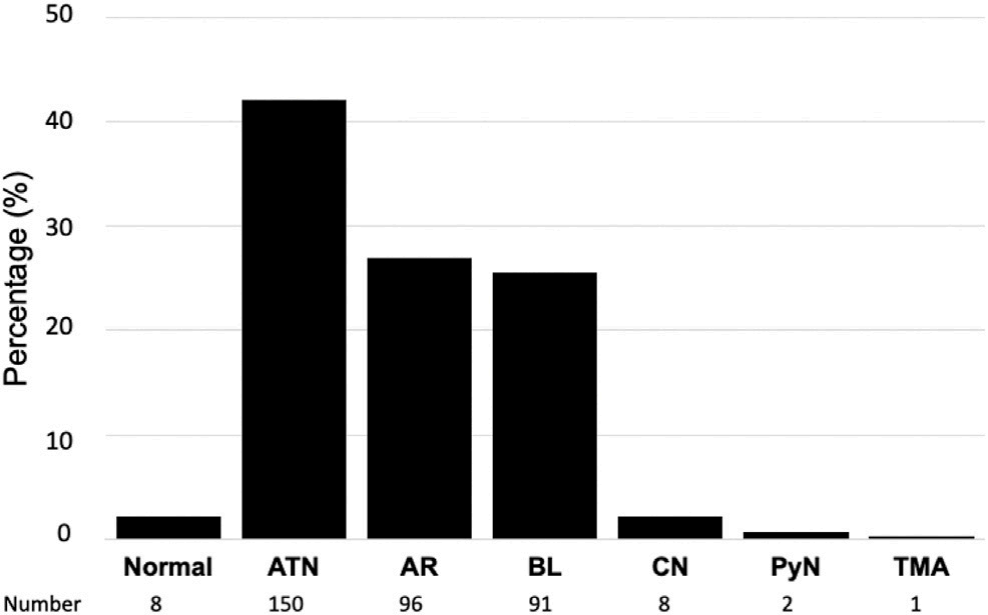
Histological diagnosis in surveillance biopsies of kidney transplant recipients with delayed graft function. ATN, acute tubular necrosis; AR, acute rejection; BL, borderline findings; CN, cortical necrosis; PyN, pyelonephritis; TMA, thrombotic microangiopathy.

The number of patients with one, two, and three biopsies was 317, 15, and 3 respectively. As shown in Figure 2, eight biopsies (2.2%) were classified as normal kidney transplant, 150 (42.1%) presented acute tubular necrosis (ATN), 91 (25.5%) presented borderline changes, 96 (26.9%) were acute rejections, either cellular (91 cases, 25.5%) or antibody-mediated (5 cases, 1.4%), 8 (2.2%) presented coagulation necrosis, 2 (0.5%) had acute pyelonephritis and one biopsy (0.2%) showed thrombotic microangiopathy (Figure 2).

**FIGURE 3:**
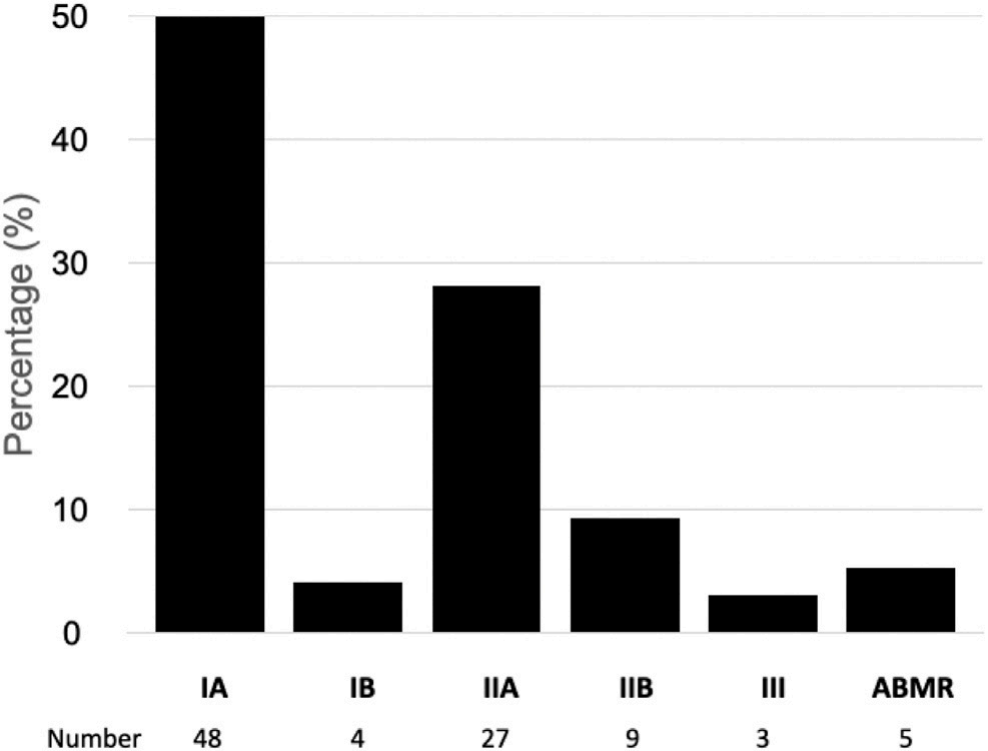
Banff classification of rejection in kidney transplant recipients with delayed graft function. ABMR, antibody mediated acute rejection.

Figure 3 shows the Banff grades of the biopsies interpreted as acute rejection. Most were cellular rejections, predominantly IA and IIA phenotypes, with a lower frequency of the more severe cellular phenotypes and antibody-mediated rejection. All biopsies with acute antibody-mediated rejection were from patients with increased immunological risk who received induction therapy with ATG (Figure 3).

Table 2 shows risk factors for acute rejection in the univariate and multivariate analysis. Positive historic B cell cross-matching (*p* = 0.023), vascular anastomosis time (*p* = 0.0001), DGF duration (*p* < 0.05), and absence of induction therapy with ATG (*p* = 0.0001) were associated with acute rejection. These risk factors along with the recipient’s age (*p* < 0.2) were included in the multivariate analysis model, which showed that the recipient’s age, DGF duration, and absence of induction therapy with ATG were significantly associated with the occurrence of acute rejection (Table 2).

**TABLE 2:** Univariate and multivariate analysis of acute rejection risk factors.

Univariate analysis	PR	95% CI	*p*-value
Donor related factors			
Age	1.004	0.993–1.015	0.474
Ethnicity (non-white)	1.040	0.659–1.641	0.865
Expanded criteria donor	1.053	0.731–1.518	0.781
Acute kidney injury	1.140	0.808–1.608	0.456
Recipient related factors			
Age	0.991	0.979–1.004	0.164
Ethnicity (non-white)	1.158	0.763–1.713	0.462
Gender (male)	1.172	0.808–1.699	0.402
Previous transplantation	0.545	0.217–1.371	0.298
Absence of induction therapy with ATG	2.140	1.422–3.221	0.000
PRA > 0	0.801	0.567–1.133	0.209
Presence of DSA	0.761	0.443–1.307	0.322
HLA mismatches	1.106	0.944–1.296	0.213
Positive historic B cell crossmatch	2.188	1.124–4.260	0.021
DGF duration	1.010	1.003–1.017	0.005
Surgery related factors			
Cold ischemia time	0.989	0.961–1.016	0.418
Vascular anastomosis time	1.018	1.009–1.027	0.000
Multivariate Analysis			
Recipient age	0.985	0.972–0.998	0.028
Absence of induction therapy with ATG	2.320	1.443–3.731	0.001
Positive historic B cell crossmatch	1.634	0.802–3.327	0.176
DGF duration	1.011	1.003–1.019	0.005
Vascular anastomosis time	1.010	0.999–1.022	0.080

PRA, panel reactive antibodies; DSA, donor specific antibody; DGF, delayed graft function; PR, prevalence ratio; 95% CI, 95% confidence interval.

### Delayed Graft Function Duration and the Occurrence of Acute Rejection

For this analysis, we divided patients and biopsies into four groups according to DGF duration. In group 1, with DGF duration up to 7 days, 44 biopsies were performed in 43 patients with eight episodes of rejection identified (18.2%). In group 2, with DGF duration between 8 and 14 days, there were 93 biopsies in 83 patients with 20 rejection episodes (21.5%). In group 3, with DGF duration between 15 and 21 days, there were 76 biopsies in 70 patients with 19 rejection episodes (25.0%) and, in group 4, with DGF duration longer than 21 days, 143 biopsies were performed in 139 patients with 49 episodes of rejection (34.3%) (*p* = 0.005). Table 3 presents the frequency of the histological diagnoses according to DGF duration. Noteworthy, the frequency of acute tubular necrosis decreased and the frequency of acute rejection increased as DGF lasted longer.

**TABLE 3:** Frequency of histological findings from surveillance biopsies of kidney transplant recipients according to DGF duration.^a^

Histological finding (Number of biopsies)	ATN	Borderline	Acute rejection	Other lesions
	(150)	(91)	(96)	(19)
DGF duration				
≤7 days (41)	22 (50%)^b^	9 (20.4%)^c^	8 (18.2%)	2 (11.4%)
8–14 days (90)	39 (41.9%)	28 (30.1%)	20 (21.5%)	3 (6.4%)
15–21 days (75)	33 (43.4%)	23 (30.3%)	19 (25.0%)	0 (0%)
≥22 days (142)	56 (39.2%)	31 (21.7%)	49 (34.3%)^d^	6 (4.9%)

a Excluding normal biopsies.

b ATN significantly higher than the other groups (*p* < 0.05).

c Borderline findings significantly lower than the other groups (*p* < 0.05).

d Acute rejection significantly higher than the other groups (*p* < 0.05).

### Immunosuppressive Regimen and Presence of Rejection in the Surveillance Biopsy

The type of immunosuppressive regimen was associated with the occurrence of acute rejection. In the group of 36 patients who did not receive antibody-induction therapy, acute rejection incidence was 36.1% (13 patients with cellular rejections). In the group of 148 patients treated with Basiliximab, the incidence was 37.8% (56 patients with cellular rejections), and, in the 151 patients who received ATG, an incidence of 17.9% of acute rejection was observed (27 patients with rejection, being 22 with cellular rejections and 5 with antibody-mediated rejections). No difference in acute rejection incidence was observed between patients treated with Baxiliximab and those without antibody-induction therapy (*p* = 0.126). However, patients treated with ATG had a significantly lower incidence of acute rejection than those in the other two groups (*p* = 0.0001).

Table 4 shows the frequency of acute rejection according to the presence and type of antibody induction therapy. The lower incidence of acute rejection in the group of patients treated with ATG occurred despite the higher risk of rejection present in this group who presented longer cold ischemia time (25:02 ± 5:54, 19:55 ± 4:57, 17:27 ± 5:13 h:min; *p* = 0.001), higher class I PRA (28.1 ± 35.6, 4.1 ± 9.7, 5.2 ± 18.2%; *p* = 0.001), higher class II PRA (24.2 ± 32.1, 2.0 ± 8.6, 5.9 ± 15.9%; *p* = 0.001), higher frequency of re-transplants (16.5, 1.3, 0%; *p* = 0.002), and more patients with donor-specific anti-HLA antibodies (30.5, 6.1, 5.5%; *p* = 0.003), respectively for the ATG, Basiliximab and no-induction groups.

**TABLE 4:** Frequency of rejection in the biopsies according to the Banff classification and antibody-induction therapy status.

Patients/Biopsies	No Ab induction	Basiliximab	ATG
	(36/42)	(148/157)	(151/157)
Banff classification			
Borderline	10 (23.8%)	46 (29.3%)	35 (22.3%)
IA	4 (9.5%)	36 (22.9%)	8 (5.1%)
IB	2 (4.7%)	2 (1.3%)	0 (0%)
IIA	6 (14.3%)	11 (7.0%)	10 (6.4%)
IIB	0 (0%)	5 (3.2%)	4 (2.5%)
III	1 (2.4%)	2 (1.3%)	0 (0%)
ABMR	0 (0%)	0 (0%)	5 (3.2%)
All rejections^a^	13 (30.9%)	56 (35.7%)	27 (17.9%)**

Ab, antibody; ABMR, antibody-mediated acute rejection; ATG, Anti Thymocyte globulin.

a Excluding borderline findings; ** = *p* < 0.05.

Table 5 shows the analyzes of the impact of antibody induction on the incidence of acute rejection in patients at lower risk for rejection. The analysis included patients without anti-HLA sensitization and patients without anti-donor HLA antibodies receiving tacrolimus and sodium mycophenolate. A high incidence of rejection, remarkably in the groups of patients that did not receive antibody-induction or received Basiliximab, occurred despite the lower risk and use of this association of immunosuppressive agents. In comparison to the group of patients treated with Basiliximab, the group of patients that received ATG had a significant reduction in the incidence of rejection.

**TABLE 5:** Incidence of acute rejection in unsensitized patients and patients without donor-specific HLA antibodies, receiving tacrolimus and sodium mycophenolate, according to antibody-induction therapy status.

Category	(Number of patients)	With/without rejection	% With rejection	*p*
0% PRA, no induction^a^	(18)	4/14	22.2	0.345 vs.^b^
0% PRA, Basiliximab^b^	(65)	25/40	38.5	0.009 vs.^c^
0% PRA, ATG^c^	(36)	4/32	11.1	0.652 vs.^a^
No DSA, no induction^d^	(19)	4/15	21.1	0.198 vs.^e^
No DSA, Basiliximab^e^	(107)	42/65	39.3	0.001 vs.^f^
No DSA, ATG^f^	(86)	10/76	11.6	0.655 vs.^d^

PRA, panel reactive antibodies; DSA, donor specific anti-HLA antibodies.

The small letters identify the groups of patients according to the presence and type of induction therapy and the respective group comparisons.

### Antibody Induction Therapy, Acute Rejection, and Patient and Graft Survival

**FIGURE 4:**
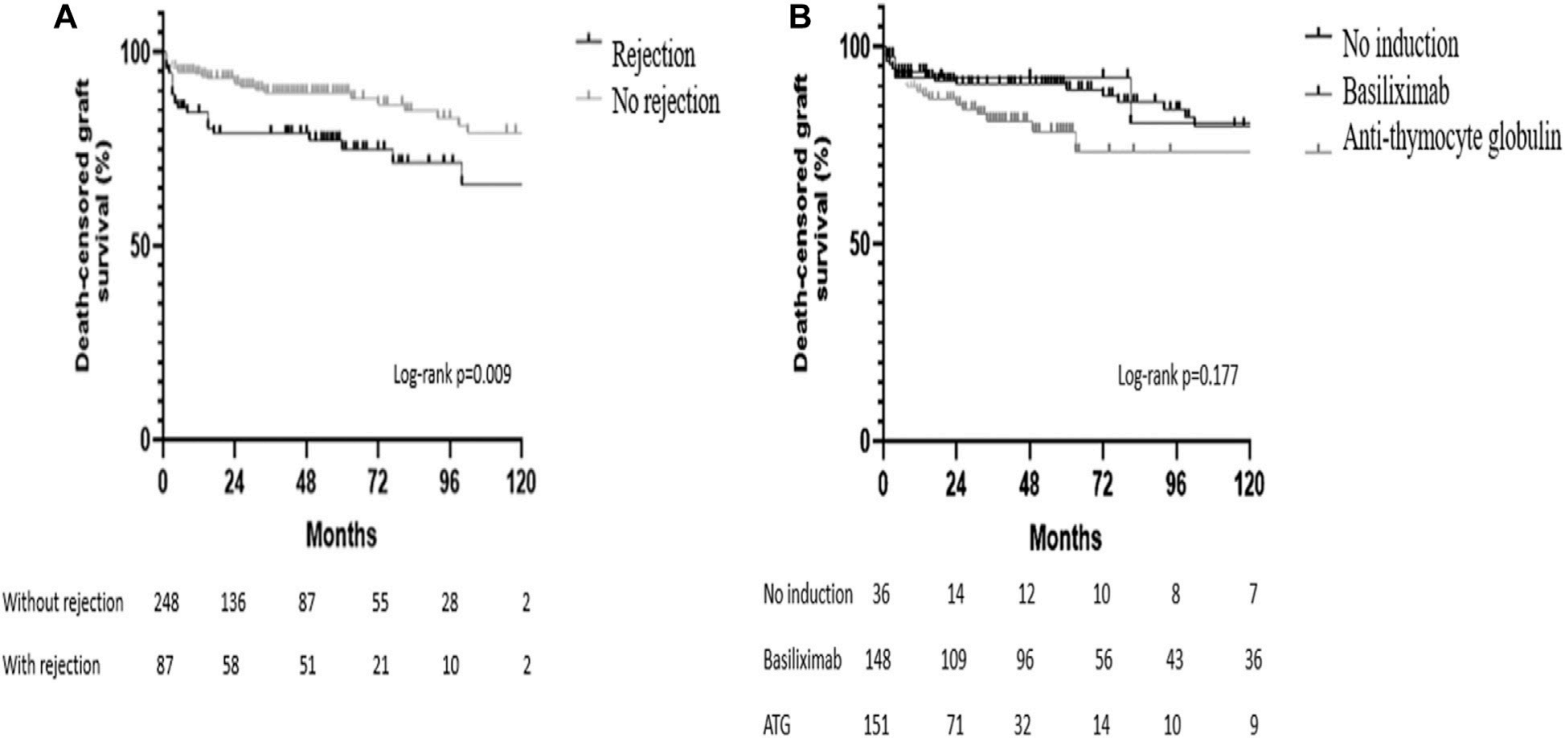
Kaplan-Meier survival curves. **(A)** Death-censored graft survival according to the occurrence of acute rejection in the surveillance biopsy; **(B)** Death-censored graft survival according to the use and type of antibody-induction therapy.

Neither acute rejection (*p* = 0.145) nor the use or type of antibody-induction therapy (*p* = 0.665) were associated with patient survival. Death censored graft survival was significantly lower in the group of patients with acute rejection (log-rank *p* = 0.009) and was not influenced by the use or type of antibody-induction therapy (log-rank *p* = 0.177) (Figure 4).

Table 6 shows risk factors for graft failure in the univariate and multivariate analysis. Recipient age (*p* = 0.003), male gender (*p* = 0.045), and DGF duration were associated with graft loss. These risk factors along with donor’s ethnicity, cold ischemia time, and acute rejection were included in the multivariate analysis model, which showed that the recipient’s age, recipient’s male gender, DGF duration, and acute rejection were significantly associated with graft loss (Table 6). To further analyze the role of DGF duration as a risk factor for graft loss, we performed a sub-analysis including only the patients who presented ATN at the surveillance biopsies and found that both mean survival time and 1-year death censored graft survival decreased in parallel to DGF duration (*p* < 0.0095).

**TABLE 6:** Univariate and multivariate analysis of graft survival risk factors.

Univariate analysis	PR	95% CI	*p*-value
Donor related factors			
Age	0.998	0.994–1.002	0.348
Ethnicity (non-white)	0.882	0.732–1.063	0.187
Expanded criteria donor	0.998	0.835–1.193	0.982
Acute kidney injury	0.955	0.844–1.080	0.461
Recipient related factors			
Age	0.993	0.989–0.998	0.003
Ethnicity (non-white)	1.083	0.904–1.297	0.386
Gender (male)	1.136	1.003–1.287	0.045
Previous transplantation	0.873	0.676–1.128	0.299
Absence of induction therapy with ATG	1.014	0.758–1.354	0.839
PRA > 0	1.013	0.908–1.130	0.819
Presence of DSA	0.992	0.846–1.162	0.917
HLA mismatches	1.045	0.977–1.117	0.203
Positive historic B cell crossmatch	0.957	0.600–1.527	0.854
DGF duration	0.987	0.982–0.992	0.000
Surgery related factors			
Cold ischemia time	1.009	0.998–1.020	0.115
Vascular anastomosis time	0.999	0.992–1.006	0.738
Acute rejection	1.125	0.994–1.274	0.062
Multivariate Analysis			
Donor ethnicity (non-white)	0.933	0.809–1.077	0.344
Recipient age	0.993	0.989–0.997	0.001
Recipient gender (male)	1.149	1.021–1.294	0.021
DGF duration	0.987	0.983–0.991	0.000
Cold ischemia time	1.007	0.998–1.017	0.127
Acute rejection	1.158	1.041–1.287	0.007

PRA, panel reactive antibodies; DSA, donor specific antibody; DGF, delayed graft function; PR, prevalence ratio; 95% CI, 95% confidence interval.

Grafts were lost due to chronic graft failure in 40 cases (2 in the no induction group, 18 in the ATG group, and 20 in the Basiliximab group), immunological causes (acute or chronic rejections) in 12 cases (none in the no induction group, 5 in the ATG group and 7 in the Basiliximab group), vascular causes in 6 cases (2 in the no induction group, none in the Basiliximab group and 4 in the ATG group) and recurrent focal and segmental glomerulosclerosis in 1 case.

## Discussion

In the present study, we analyzed the histological findings on surveillance graft biopsies of kidney transplant recipients experiencing DGF. The main findings were acute tubular necrosis and a high incidence of other graft injuries occurred, particularly acute cellular rejection and borderline findings. We also found that the occurrence of acute rejection during DGF leads to inferior graft survival.

Delayed graft function is a frequent complication of brain-dead deceased donor kidney transplantation and is even more frequent in kidney transplants from donors on circulatory death (20). Kidney transplantation from expanded criteria donors and/or high KDPI donors is also associated with a high incidence (13, 21). Clinically DGF presents as a post-transplant acute kidney injury with significantly increased serum creatinine and many times with decreased urinary output leading to the necessity of renal replacement therapy. Among other factors, it may be associated with organ procurement characteristics (inotropic support of the donor, cold ischemia time, and cold storage preservation), donor characteristics (age, renal function, and comorbidities), and recipient characteristics (hypovolemia, previous transplantation, preformed anti-donor antibodies and obesity). The transplant surgery itself and postoperative care (hydration, vascular anastomosis time, and hemodynamic support) can also influence the occurrence of DGF (12, 22-25).

The incidence of DGF is highly variable worldwide. It varies from around one-fourth to approximately two-thirds of kidney transplants from brain-dead deceased donors (26–29). In Brazil, for reasons that are not entirely understood, the reported incidence of DGF is consistently higher compared to other registries (7-9). In a recently published large Brazilian multicenter study, we found a high incidence of DGF and concluded that late referral and poor donor maintenance account for the high overall incidence while variability in donor and recipient selection, organ preservation method, and type of antibody induction may account for the wide variation observed among centers (7).

Two-thirds of the recipients in the present study developed DGF by the definition adopted in the present study. This frequency is similar to the one found in the Brazilian multicenter study (7) and to previous monocentric Brazilian studies (8, 9) but is much higher than that reported in other national registries (28, 29). Importantly, we observed a high incidence of acute rejection and borderline findings in the surveillance biopsies, which is in line with previous reports (8, 9). Additionally, in agreement with a large study in the Australian and New Zealand Dialysis and Transplant Registry database, we found that longer periods of DGF correlated with a higher incidence of rejection (29).

As expected, the largest percentage of the biopsies obtained during the DGF period presented with a histological diagnosis of acute tubular necrosis. However, Banff grade IA or higher acute rejections occurred in one-fourth of the biopsies. A noteworthy overtime change occurred in the frequency of the histological lesions. The frequency of acute tubular necrosis decreased, and the frequency of rejection increased, reflecting the healing of ischemia and reperfusion injuries partially replaced by alloimmune injury, possibly acting for maintaining graft dysfunction. Vascular anastomosis time was highly significant at the univariate analysis but presented borderline statistical value in the multivariate analysis. In line with our findings, a recent publication by Lim et al. reported an important association between DGF duration and acute rejection. They described that three-quarters of the acute rejection episodes occurred in kidney transplant recipients whose DGF lasted longer than 2 weeks (29). Moreover, borderline findings were diagnosed in another one-fourth of the surveillance biopsies. Such histological finding may be the expression of an initial T cell-mediated rejection but may also be due to non-alloimmune inflammation induced by different injuries, leaving its significance uncertain. This often represents a treatment dilemma for the transplant physician, particularly in non-functioning allografts, as in DGF (14, 30). In our cohort half of the patients with borderline findings were treated for rejection.

Some controversy remains on whether or not DGF is associated with an increased incidence of acute rejection and reduced graft survival. Such may be due to a lack of homogeneity of the study cohorts and the non-uniform DGF definitions (5, 6, 14, 17). However, several studies describe a higher incidence of rejection in kidney transplant recipients experiencing DGF (5, 14, 17). Wu and collaborators reported that DGF is a major risk factor for acute rejection in the modern era of immunosuppression in deceased donor kidney transplants, showing that the cumulative probability for rejection was greater in patients undergoing DGF at all points during the follow-up period (17). Also, a recent study by Weber et al. demonstrated that the hazard ratio for developing acute rejection within the first year after transplantation was 71% higher in the group of patients with DGF (14). The impact of DGF on graft survival is well established, as shown in a recently published large multicenter study (7). However, controversy is still out on whether the worst graft survival is restricted only to recipients of kidneys from standard criteria donors (31, 32).

Perhaps the major hurdle of DGF is the inability to detect or even suspect the occurrence of acute rejection due to the lack of functional parameters usually used to monitor injuries. Importantly, in the absence of accurate non-invasive methods or biomarkers of rejection in this setting, the only reliable tool to uncover alloimmune graft injury is the surveillance graft biopsy.

In agreement with previous studies, our results demonstrated that the rejection rate decreases with age supporting the notion that immunosuppressive therapy may be reduced in elderly recipients due to the progressive decline in immune functions, leading to a lower risk of rejection and a higher risk of infectious complications (33–35).

Antibody induction therapy with polyclonal anti-T cell antibodies is often used to prevent acute rejection, particularly in recipients with high immunological risk. In our study, the group of patients who received ATG was at higher immunological risk and, despite this, had a substantially lower incidence of rejection in the surveillance biopsy as compared to patients that received monoclonal anti-IL2 receptor antibodies or patients who did not receive antibody-induction therapy. Recently, Alloway and collaborators reported a reanalysis of the data from prior trials comparing ATG with anti-IL-2 receptor monoclonal antibodies, showing the superiority of such polyclonal antibodies in preventing acute rejection (36). Moreover, in previous studies including patients with DGF, ATG was more efficient in preventing acute rejection (37, 38).

Interestingly a considerably high incidence of histological rejection occurred even in patients considered of lower immunological risk such as patients without anti-HLA sensitization, and patients without anti-donor HLA antibodies, receiving ATG induction and baseline immunosuppressive regimen with steroids, tacrolimus, and sodium mycophenolate. In the group of patients that did not receive antibody induction and in the group that received Basiliximab the incidence of rejection was very high despite the potent association of baseline immunosuppressive agents (39). These findings give support to the notion that ischemia and reperfusion injury, by overexposing graft antigens, elicits a strong alloimmune response (12, 40).

A study by Hatoum and collaborators evaluated the utility of surveillance allograft biopsies during DGF in patients receiving antibody-induction therapy with ATG or Basiliximab and baseline immunosuppression with steroids, mycophenolate, and tacrolimus. They concluded that rejection episodes during DGF are uncommon and, therefore, the usefulness of serial surveillance biopsies is limited. These results differ from ours in some ways, including a much lower incidence of DGF, the inclusion of kidney recipients of living donors, and a higher proportion of African-American recipients (38). Therefore, the differences found in the incidence of rejection are probably due to the study population, sample sizes, and severity of the ischemia-reperfusion injury.

It is conceivable that with a higher incidence of DGF and the use of organs from expanded criteria donors, the incidence of rejection would be higher (14). Importantly our study occurred within a period in which the donor acceptance policy and immunosuppressive regimen did not change substantially. However, current immunosuppressive regimens are different from those employed at the time of guidelines publications. Nevertheless, even under current immunosuppressive regimens a high frequency of histological lesions, mainly acute rejections, are uncovered by surveillance biopsies.

A recent study by Van Loon et al. also revealed a very high incidence of acute rejection in their subset of patients with DGF. In their study, the risk factors for rejection were HLA mismatches and pre-transplant HLA-DSA. The authors found that non-immune risk factors were not strong risk factors for early inflammation (41). This is in contrast with our findings where the recipient age, DGF duration, and absence of antibody-induction with polyclonal anti-T cell antibodies were the identified risk factors. We believe that the discrepancies may be related, at least in part, to the frequency and type of antibody-induction therapy since in our more patients received antibody-induction with ATG.

The association between prolonged vascular anastomosis time and DGF is long known and was confirmed in two recent studies (26, 27). However, a possible association between prolonged vascular anastomosis time and occurrence of acute rejection, as seen in this study, is new and deserves further investigation. Prolongation of warm ischemia time can lead to more intense ischemia and reperfusion injury. A well-known consequence of such injury is the activation of innate immunity signaling transcription factors that encode genes involved in the regulation of inflammation. In such an inflamed environment, graft antigens are more exposed, and therefore recognized and processed by antigen-presenting cells and presented to the host immune system, facilitating the occurrence of acute rejection (41, 42).

The present work, by selecting only biopsies of patients with DGF, does not allow the analysis of DGF risk factors, particularly cold ischemia time. However, in previous studies, cold ischemia time surfaced as an independent risk factor for acute rejection (24, 43). We did not find such correlation and believe that a possible effect may have been lost due to the usually prolonged cold ischemia time, observed in our region, and perhaps for sample size matters.

Our study has limitations intrinsic to its retrospective and monocentric design. Early protocol biopsies of function kidney grafts, at a similar time as the surveillance biopsies in DGF, could also reveal unsuspected lesions. We did not analyze the outcomes of patients who had DGF but were not subjected to the surveillance biopsy. Also, caution is needed in the interpretation of the immunosuppressive regimen results due to the non-randomized design. Nevertheless, the timing of the biopsies was dictated by current guidelines and, a considerable incidence of acute cellular rejection occurred during the DGF period indicating that surveillance biopsies are instrumental for the clinical care of kidney transplant recipients.

In conclusion, the surveillance biopsy of kidney grafts with DGF remains an essential tool for the clinical care of the kidney transplant recipient. These biopsies are even more important in settings where kidneys from expanded criteria donors and/or high KPDI donors are frequently utilized, with prolonged cold ischemia time and high incidence of DGF.

## Data Availability

The original contributions presented in the study are included in the article/supplementary material, further inquiries can be directed to the corresponding author.
